# The concept of Cheeger deformations on fiber bundles with compact structure group

**DOI:** 10.1007/s40863-022-00343-7

**Published:** 2022-11-10

**Authors:** Leonardo F. Cavenaghi, Lino Grama, Llohann D. Sperança

**Affiliations:** 1 Instituto de Matemática, Estatística e Computação Cinetífica – Unicamp, Rua Sérgio Buarque de Holanda, 651, Campinas, SP 13083-859 Brazil; 2 Instituto de Ciência e Tecnologia – Unifesp, Avenida Cesare Mansueto Giulio Lattes, 1201, São José dos Campos, SP 12247-014 Brazil

**Keywords:** Cheeger deformantions, Fiber bundles, Non-negative curvatures, Fat bundles, Positive Ricci, Scalar and sectional curvatures

## Abstract

The purpose of this paper is two-fold: we systematically introduce the notion of Cheeger deformations on fiber bundles with compact structure groups, and recover in a very simple and unified fashion several results that either already appear in the literature or are known by experts, though are not explicitly written elsewhere. We re-prove: Schwachhöfer–Tuschmann Theorem on bi-quotients, many results due to Fukaya and Yamaguchi, as well as, naturally extend the work of Searle–Solórzano–Wilhelm on regularization properties of Cheeger deformations, among others. In this sense, this paper should be understood as a survey intended to demonstrate the power of Cheeger deformations. Even though some of the results here appearing may not be known as stated in the presented form, they were already expected, being our contribution to the standardization and spread of the technique via a unique language.

## Introduction

The metric deformation known as *Cheeger deformations* was firstly introduced on [2]. Its main goal was to produce metrics with non-negative sectional curvature on manifolds with symmetries. Since then, Cheeger deformations were used in [9, 11] to produce new examples of manifolds with non-negative and positive sectional curvature; in [13, 19, 20] to study curvature properties on homogeneous spaces such as biquotients, on [21] to lift positive Ricci curvature from a metric quotient *M*/*G* to *M*, and in [4, 5, 11] to provide examples of manifolds with non-negative sectional and/or positive Ricci curvatures. Other interesting results along the same lines are in [1, 8, 12, 19]. Here we introduce an analogous metric deformation, defined on a specific class of metrics on fiber bundles with compact structure groups, naturally supported on Cheeger deformations. Throughout the manuscript we provide other references, including results on the existence metrics of *almost non-negative sectional curvature*.

Recall that from any fiber bundle $F \hookrightarrow M \rightarrow B$ with compact structure group *G* can be decoupled a principal *G*-bundle $\mathcal P \rightarrow B$ and a manifold *F* with an effective *G*-action. Here, *M* can be recovered via a submersion $\overline{\pi }: \mathcal P \times F \rightarrow M$ with fiber *G*. The idea of our deformation consists of inducing a one parameter family of metrics on *M* via $\overline{\pi }$ after making Cheeger deformation on $\mathcal P$. That is, if $\textit{g}$ is a Riemannian metric on $\mathcal P$ for which *G* acts via isometries, given any *G*-invariant metric $\textit{g}_F$ on *F* we look to the metric $\textit{h}_t$ on *M* obtained from $\textit{g}_t + \textit{g}_F$ in $\mathcal P \times F$, see Definition 1 for further details.

All the long we mostly follow the approach in [25] and [16], introducing useful tensors to standardize the analysis of this deformation, such as nowadays well established the basics on Cheeger deformations. As a very useful formula we shall obtain:

Let $\textit{h}$ obtained via $\overline{\pi }: (\mathcal P \times F, \textit{g}+ \textit{g}_F) \rightarrow (M,\textit{h})$ and let $\textit{g}_t$ be a Cheeger deformation of $\textit{g}$. Then, for every pair ${\tilde{X}} = X + X_F + U^*$, ${\tilde{Y}} = Y + Y_F + V^*$ of tangent vectors to *M*, appropriately decomposed, it holds that

1 $$\begin{aligned} {\tilde{\kappa }}_{t}\left( {\tilde{X}}, {\tilde{Y}}\right) = \kappa _t \left( X{+}U^{\vee },Y{+}V^{\vee }\right) {+} K_{\textit{g}_F}\left( X_F - \left( P_F^{-1}PU \right) ^*, Y_F - \left( P_F^{-1}PV \right) ^*\right) + {\tilde{z}}_t \left( {\tilde{X}},{\tilde{Y}}\right) , \end{aligned}$$

where ${\tilde{\kappa }}_t$ is the unreduced sectional curvature of the metric $\textit{h}_t$ computed in an appropriate reparametrization of the plane ${\tilde{X}}\wedge {\tilde{Y}}$, $\kappa _t$ is the unreduced sectional curvature of $\textit{g}_t$ and $K_{\textit{g}_F}$ is the unreduced sectional curvature of $\textit{g}_F$. Moreover, ${\tilde{z}}_t$ is a non-negative term.

We stress it out that it is not of the author’s knowledge whether Eq. (1) already appears elsewhere in such a general manner. However, when collapsing the fiber *F* to a single point, it naturally yields to the well known expression of the sectional curvature of a Cheeger deformation computed at some reparameterized planes, see [25, Proposition 1.3, p.2] or Eq. (5).

Taking advantage of Eq. (1), we re-prove in a very general picture results on almost non-negative sectional curvature appearing in [7]. Such results as stated were either already known (see [25]) or expected to be true, though not explicitly written elsewhere.

### Theorem 1.1

(Fukaya–Yamaguchi) Let $F \hookrightarrow M \rightarrow B$ be a bundle with compact structure group *G*, fiber *F* and base *B*. Assume that *M* is an associate bundle to $\pi : (\mathcal P,\textit{g}) \rightarrow B$ such that:

1. $$K_{\textit{g}}\ge 0$$;
2. *F* has a *G*-invariant metric $\textit{g}_F$ of non-negative sectional curvature.

*Then* *M* *admits a sequence of Riemannian metrics* $\{\textit{g}_n\}$ *such that* $\mathrm {sec}_{\textit{g}_n} \ge -\frac{1}{n},$ $\mathrm {diam}~(M,\textit{g}_n) \le \frac{1}{n}.$

A Fukaya–Yamaguchi type result on the existence of almost non-negative Ricci curvature, namely:

### Theorem 1.2

Let $F\hookrightarrow M \rightarrow B$ be a fiber bundle with compact structure group *G* and total space *M*. Also assume that *F* carries a metric $\textit{g}_F$ of non-negative Ricci curvature and *B* carries a metric $\textit{g}_{\epsilon }$ with ${\mathrm{Ric}}(\textit{g}_{\epsilon }) \ge -\epsilon ^2$. Then *M* carries a metric $\textit{h}_{\epsilon }$ with ${\mathrm{Ric}}(\textit{h}_{\epsilon }) \ge -\epsilon ^2$.

Both Theorems 1.1 and 1.2 should follow from the computations in [19], though these follow very directly from our techniques. We also reinforce that Theorem 1.2 was first conjectured to be true in [7, Conjecture 0.14, p.257], see also [3, 22, 24]. Notably as well is the fact that this kind of result is of interest in the field of Metric Geometry, though we do not touch this area here, being the above-mentioned theorem proofs of concept to the deformation here developed. Other very useful references related to these subjects are: [15, 23].

All the analyses coming out from Eq. (1) allows us to recover in a very simple fashion classical results in bi-quotients, such as:

### Theorem 1.3

(Schwachhöfer–Tuschmann) Any bi-quotient *G*//*K* from a compact Lie group *G* admits a metric with positive Ricci curvature and almost non-negative sectional curvature simultaneously if, and only if, *G*//*K* has finite fundamental group.

Finally, we obtained two further applications. Recall for instance that in [18] Searle–Solórzano–Wilhelm show that Cheeger deformations work as a strong regularization process: appropriate scaling of the family of metrics on Cheeger deformations imply $C^p$-convergence, for any $p\ge 0$ a priori fixed, to metrics with totally geodesic fibers. We apply this idea here to prove:

### Theorem 1.4

Let $\pi : F\hookrightarrow M \rightarrow B$ be a fiber bundle with compact total space and compact structure group *G*. Assume that $\textit{h}$ is a Riemannian submersion metric on *M* obtained via the submersion $\overline{\pi }:  (\mathcal P\times F,\textit{g}+\textit{g}_F) \rightarrow M$, where $\mathcal P$ is the associated principal bundle to $\pi$ and $\textit{g}, \textit{g}_F$ are, respectively, *G*-invariant metrics on $\mathcal P$ and *F*. Then, for any integer $p\ge 0$, after an appropriate re-scaling the fibers of $\pi$, the metric deformation $\textit{h}_t$ (Definition 1, Sect. 3), converges in the $C^p$-topology to a Riemannian submersion metric with totally geodesic fibers.

Theorem 1.4 was already expected to be true but more importantly, this regularization nature of Cheeger deformations already appears in the proof of all the mentioned results in this intro. As it is also clear, the sectional curvature formulae (1) may lead to new conjectures in which concerns the existence of metrics with positive sectional curvature on the total space of some fiber bundles.

Recall, for instance, the fiber dimension Petersen–Wilhelm conjecture:

### Conjecture A

(Petersen–Wilhelm Fiber dimension conjecture). If $F\hookrightarrow M\rightarrow B$ is a Riemannian submersion from a positively curved closed manifold *M*, then

$$ \dim F<\dim B.$$

In Sect. 5 we make some comments on this conjecture in the case of fiber bundles with the structure group being $S^3, SO(3)$. More precisely, we conjecture:

### Conjecture B

(Principal bundle Strong Petersen–Wilhelm conjecture). Any $S^3, SO(3)$ principal bundle over a positively curved manifold admits a metric with positive sectional curvature if, and only if, such a submersion is fat.

Assuming the validity of Conjecture B it shall be straightforward to check that: Any $S^2\hookrightarrow M \rightarrow B$ fat bundle with structure group *SO*(3) admits a metric of non-negative sectional and positive vertizontal curvature. In particular, $\dim B\ge 4$.

### Notation and conventions

We denote by $R_{\textit{g}}$ the Riemannian tensor of the metric $\textit{g}$:

$$\begin{aligned} R_{\textit{g}}(X,Y)Z=\nabla _X\nabla _YZ-\nabla _Y\nabla _XZ-\nabla _{[X,Y]}Z, \end{aligned}$$

where $\nabla$ stands for the Levi-Civita connection of $\textit{g}$. We denote either by $K_{\textit{g}}(X,Y)=\textit{g}(R_{\textit{g}}(X,Y)Y,X)$ or by $R_{\textit{g}}(X,Y),$ making it clear in the context, the unreduced sectional curvature of $\textit{g}$. The Ricci tensor of $\textit{g}$ is defined by

$$\begin{aligned} {\mathrm{Ric}}_{\textit{g}}(X,Y)=\sum _{i=1}^ng(R(e_i,X)Y,e_i), \end{aligned}$$

where $\{e_1,...,e_n\}$ is an orthonormal basis for $\textit{g}$. The associated quadratic form is denoted by ${\mathrm{Ric}}_{\textit{g}}(X)={\mathrm{Ric}}_{\textit{g}}(X,X)$.

Whenever we say we have a *Riemannian principal bundle* we mean that the principal bundle is considered with a Riemannian submersion metric.

## (Classical) Cheeger deformations

We first recall the procedure known as *Cheeger deformations*. Though the main formulae come from classical references, such as [25] and [16], we shall proceed differently in which concerns the presentation. The reason for that is to make more natural our definition of Cheeger deformation on fiber bundles.

Take the product manifold $M\times G$ with the product metric $\textit{g}+t^{-1}Q$, where *G* acts on *M* via isometries and *Q* is a bi-invariant metric on *G*. We therefore see ourselves with two possibilities of free (and commuting) actions:2In (2) the action $\bullet$ stands to

3 $$\begin{aligned} r\bullet (m,g) := (m,rg), \end{aligned}$$

while the action $\star$ is nothing but the associated bundle action on $M\times G$, that is

4 $$\begin{aligned} r\star (m,g) := (rm,rg). \end{aligned}$$

Therefore, $\pi ((m,g)) := m$ meanwhile $\pi '((m,g)) := g^{-1}m$. Since $\pi$ and $\pi '$ define principal bundles, the metric $\textit{g}+t^{-1}Q$ induces via $\pi$ and $\pi '$, respectively, the metrics $\textit{g}$ (the original one) and $\textit{g}_t$, a *Cheeger deformation* of $\textit{g}$. Also note that although $\textit{g}_1 = \textit{g}$, the horizontal space obtained via $\pi '$ has a different angular position in relation to the horizontal space obtained via $\pi$, what can be directly checked from the fact that $\textit{g}_1(\cdot , \cdot ) = \textit{g}(C_1\cdot ,\cdot ) = \textit{g}((1+P)^{-1}\cdot ,\cdot ).$

Throughout the paper it is shall be denoted by $\mathfrak {m}_p$ the *Q*-orthogonal complement of $\mathfrak {g}_p$, the Lie algebra of $G_p$. We recall that $\mathfrak {m}_p$ is isomorphic to the tangent space to the orbit *Gp* via *action fields*: for any $U\in \mathfrak {g}$ the corresponding action field out of *U* is defined by the rule

$$\begin{aligned} U^*_p=\frac{d}{dt}\Big |_{t=0}e^{tU}p. \end{aligned}$$

It is straightforward to check that the map $U\mapsto U^*_p$ is a linear morphism whose kernel is $\mathfrak {g}_p$. This manner, any vector tangent to $T_pGp$ is said to be *vertical*, hence, such a space is named as the *vertical space* at *p*, being denoted by $\mathcal {V}_p$. For each $p\in M$ its orthogonal complement, denoted by $\mathcal H_p$, is named *horizontal space*. A tangent vector $\overline{X} \in T_pM$ can be uniquely decomposed as $\overline{X} = X + U^{*}_p$, where *X* is horizontal and $U\in \mathfrak {m}_p$.

To more feasible to understand the geometric properties of Cheeger deformations, next we shall define useful tensors associated with Cheeger deformations, see [25] for further clarifications.

1. The *orbit tensor* at *p* is the linear map $P : \mathfrak {m}_p \rightarrow \mathfrak {m}_p$ defined by
  $$\begin{aligned} \textit{g}(U^{*},V^{*}) = Q(PU,V),\quad \forall U^{*}, V^{*} \in \mathcal {V}_p \end{aligned}$$
2. For each $t > 0$ we define $P_t:\mathfrak {m}_p\rightarrow \mathfrak {m}_p$ as
  $$\begin{aligned} \textit{g}_t(U^{*},V^{*}) = Q(P_tU,V), \quad \forall U^{*}, V^{*} \in \mathcal {V}_p \end{aligned}$$
3. The *metric tensor of* $\textit{g}_t$, $C_t:T_pM\rightarrow T_pM$ is defined as
  $$\begin{aligned} \textit{g}_t \left( \overline{X},\overline{Y}\right) = \textit{g} \left( C_t\overline{X},\overline{Y}\right) , \quad \forall \overline{X}, \overline{Y} \in T_pM \end{aligned}$$

All the three tensors above are symmetric and positive definite. The next proposition shows how they are related to each other and to the original metric quantities.

### Proposition 2.1

(Proposition 1.1 in [25]) The tensors above satisfy:

1. $$P_t = \left( P^{-1} + t1 \right) ^{-1} = P(1 + tP)^{-1}$$,
2. If $$\overline{X} = X + U^{*}$$ then $$C_t \left( \overline{X} \right) = X + \left( \left( 1 + tP \right) ^{-1}U \right) ^{*}$$.

It worth pointing it out that as first observed by Cheeger and playing a vital role in [16], the metric tensor $C_t^{-1}$ can be used to define a very informative reparametrization of 2-planes to the computation of sectional curvature. Indeed, using this reparametrization we can observe that Cheeger deformations do not create ‘new’ planes with zero sectional curvature, meaning that

### Theorem 2.2

Let $\overline{X} = X + U^{*},~ \overline{Y} = Y + V^{*}$ be tangent vectors. Then $\kappa _t \left( \overline{X},\overline{Y} \right) := R_{\textit{g}_t}\left( C_t^{-1}\overline{X},C_t^{-1}\overline{Y},C_t^{-1}\overline{Y},C_t^{-1}\overline{X}\right)$ satisfies

5 $$\begin{aligned} \kappa _t \left( \overline{X},\overline{Y}\right) = \kappa _0 \left( \overline{X},\overline{Y}\right) +\frac{t^3}{4}\Vert [PU,PV]\Vert _Q^2 + z_t \left( \overline{X},\overline{Y}\right) , \end{aligned}$$

where $z_t$ is non-negative.

We refer to either [25, Proposition 1.3] or [6, Lemma 3.5] for the details on the proof and more references. Also, with the aim of concluding this section, next we recall a formula for the Ricci curvature of Cheeger deformed metric (see also [6, Lemma 2.6, p.7)]).

### Ricci curvature

Let $\{v_1,\ldots ,v_k\}$ be a *Q*-orthonormal basis of eigenvectors of $P : \mathfrak {m}_p \rightarrow \mathfrak {m}_p$, with eigenvalues $\lambda _1\le \cdots \le \lambda _k$. Given a $\textit{g}$-orthonormal basis $\{e_{k+1},\ldots ,e_n\}$ for $\mathcal H_p$, we fix the $\textit{g}$-orthonormal basis $\{e_1,\ldots ,e_k,e_{k+1},\ldots ,e_n\}$ for $T_pM$, where $e_i = \lambda _i^{-1/2}v^{*}_i$ for $i\le k$.

The follow claim can be straightforwardly checked:

#### Claim 1

The set $\{C_t^{-1/2}e_i\}_{i=1}^n$ is a $\textit{g}_t$-orthonormal basis for $T_pM$. Moreover, $C_t^{-1/2}e_i = (1+t\lambda _i)^{1/2}e_i$ for $i \le k$ and $C_t^{-1/2}e_i = e_i$ for $i > k.$

Define the horizontal Ricci curvature as

6 $$\begin{aligned} {\mathrm{Ric}}^{\mathbf{h}} \left( \overline{X}\right) := \sum _{i=k+1}^nR \left( \overline{X},e_i,e_i,\overline{X} \right) . \end{aligned}$$

#### Lemma 1

For $\{e_1,...,e_n\}$ as above,

7 $$\begin{aligned} {\mathrm{Ric}}_{\textit{g}_t}\left( \overline{X}\right)&= {\mathrm{Ric}}_{\textit{g}}^{\mathbf{h}}\left( C_t\overline{X}\right) + \sum _{i=1}^nz_t \left( C_t^{1/2}e_i,C_t\overline{X}\right) \nonumber \\&\quad + \sum _{i=1}^k\frac{1}{1+t\lambda _i}\Big (\kappa _0(\lambda _i^{-1/2}v^{*}_i,C_t\overline{X}) + \frac{\lambda _it}{4}\Vert [v_i,tP(1+tP)^{-1}\overline{X}_\mathfrak {g}]\Vert _Q^2\Big ). \end{aligned}$$

Moreover,

8 $$\begin{aligned} {\mathrm{Ric}}_{\textit{g}_t}\left( \overline{X}\right) = {\mathrm{Ric}}_{\textit{g}}^{\mathbf{h}}\left( C_t\overline{X}\right) + \sum _{i=1}^k\frac{1}{4}\Vert [v_i,U]\Vert _Q^2 + \lim _{t\rightarrow \infty }\sum _{i=1}^nz_t \left( C_t\overline{X},C_t^{1/2}e_i \right) . \end{aligned}$$

In particular, if the action *G* is free and $\bar{\textit{g}}$ denotes the orbital distance metric in *M*/*G* it holds that

9 $$\begin{aligned} \lim _{t\rightarrow \infty }{\mathrm{Ric}}_{\textit{g}_t}(X) = {\mathrm{Ric}}_{\overline{\textit{g}}}(d\pi X) \end{aligned}$$

#### Proof

A straightforward computation following Eq. (5) gives

$$\begin{aligned} {\mathrm{Ric}}_{\textit{g}_t}\left( C_t^{-1}\overline{X}\right)&= \sum _{i=1}^{n}R_{\textit{g}_t}\left( C_t^{-1/2}e_i,C_t^{-1}\overline{X},C_t^{-1}\overline{X},C_t^{-1/2}e_i \right) =\sum _{i=1}^n\kappa _t \left( C_t^{1/2}e_i,\overline{X}\right) \\&= \sum _{i=1}^n\kappa _0 \left( C_t^{1/2}e_i,\overline{X}\right) + \sum _{i=1}^nz_t \left( C_t^{1/2}e_i,\overline{X}\right) + \frac{t^3}{4}\sum _{i=1}^k\Vert [PC_t^{1/2}\lambda _i^{-1/2}v_i,P\overline{X}_\mathfrak {g}]\Vert _Q^2 \\&= {\mathrm{Ric}}_{\textit{g}}^{\mathbf{h}}(\overline{X}) + \sum _{i=1}^nz_t(C_t^{1/2}e_i,\overline{X}) + \sum _{i=1}^k\frac{1}{1+t\lambda _i}\Big (\kappa _0(\lambda _i^{-1/2}v^{*}_i,\overline{X}) + \frac{\lambda _it^3}{4}\Vert [v_i,P\overline{X}_\mathfrak {g}]\Vert _Q^2 \Big ). \end{aligned}$$

Equations (7), (8) now follows by replacing $\overline{X}$ by $C_t\overline{X}$. Finally, Eq. (9) is derived from Lemma 4.2 in [6]. $\square$

We finish this section giving a characterization to the $z_t$-term. This shall bring more clarity to the content of Lemma 3 ahead. See also [6, Lemma 3.5, p. 11].

#### Lemma 2

It holds that

10 $$z_{t} \left( {\bar{X},\bar{Y}} \right) = 3t\max _{{\begin{array}{*{20}c}    {Z \in {\mathfrak{g}}}  \\    {|Z|_{Q}  = 1}  \\   \end{array} }} \frac{{\left\{ {dw_{Z} \left( {\bar{X},\bar{Y}} \right) + \frac{t}{2}Q\left( {[PU,PV],Z} \right)} \right\}^{2} }}{{tg(Z^{*} ,Z^{*} ) + 1}},$$

where $dw_Z$ is defined as:

11 $$\begin{aligned} w_Z : TM&\rightarrow \mathbb {R} \end{aligned}$$

12 $$\begin{aligned} \overline{X}&\mapsto \textstyle \frac{1}{2}\textit{g}(\overline{X},Z^*), \end{aligned}$$

where $Z^*$ is the action vector associated to $Z\in \mathfrak {g}$.

Moreover, if $q\in M^{reg}$, $X,Y\in \mathcal H_q$ and $U\in \mathfrak {g}$, then

13 $$\begin{aligned} dw_Z(U^{*},X)&= \frac{1}{2}X\textit{g}(U^*,Z^*) \end{aligned}$$

14 $$\begin{aligned} dw_Z(X,Y)&=-\frac{1}{2}\textit{g}([X,Y]^{\mathcal {V}},Z^*) = -\textit{g}(A_XY,Z^*). \end{aligned}$$

Therefore,

15 $$\begin{aligned} z_t(\overline{X},\overline{Y}) = 3t\left| (1+tP)^{-1/2}P\nabla ^{\mathbf {v}}_{\overline{X}}\overline{Y} - (1+tP)^{-1/2}t\frac{1}{2}[PU,PV]\right| _Q^2. \end{aligned}$$

#### Proof

We begin observing that Eqs. (13) and (14) follow from the definition of exterior derivative of 1-forms:

$$\begin{aligned} d\omega (X,Y) := X\omega (Y) - Y\omega (X) - \omega ([X,Y]). \end{aligned}$$

To continue, let us make a small digression.

#### Claim 2

Let $pr : (M,\textit{g}) \rightarrow (M/G,\overline{\textit{g}})$ be a Riemannian submersion and let *X*, *Y* be horizontal vector fields. Then

$$\begin{aligned} |A_{X}Y|_{\textit{g}}^2 = \max _{Z \in \mathfrak {g}, |Z| = 1}\left\{ dw_Z(X,Y)^2{\textit{g}}(Z^*,Z^*)^{-1}\right\} . \end{aligned}$$

#### Proof

This follows from the fact that for any vector space with inner product:

If *V* is a vector space with inner product $\langle \cdot ,\cdot \rangle$, then

$$\begin{aligned} \langle v,v\rangle = \max _{x \in V-\{0\}}\langle x,v\rangle ^2\langle x,x\rangle ^{-1}. \end{aligned}$$

Hence,

$$\begin{aligned} |A_{X}Y|_{\textit{g}}^2&= \max _{Z \in \mathfrak {g}-\{0\}}\left\{ \textit{g}(A_XY,Z^*)^2\textit{g}(Z^*,Z^*)^{-1}\right\} ,\\&= \max _{Z\in \mathfrak {g}, |Z| = 1}\left\{ dw_Z(X,Y)^2\textit{g}(Z^*,Z^*)^{-1}\right\} . \end{aligned}$$

$$\square $$

Back to the proof, we now apply Claim 2 to the Riemannian submersion $\pi ': (M\times G,\textit{g}+ \frac{1}{t}Q)\rightarrow (M,\textit{g}_t)$ since $z_t(\overline{X},\overline{Y})$ is precisely $3|A^{\pi '}_{\mathcal L_{\pi '}C_t^{-1}\overline{X}}\mathcal L_{\pi '}C_t^{-1}\overline{Y}|_{\textit{g}+ t^{-1}Q}^2$.

Denote by $\overline{w}_Z^t$, $w_Z$ and $\overline{w}_Z$ the auxiliary 1-forms (see Eq. 12) associated to the actions defined in $(M\times G,\textit{g}+ \frac{1}{t}Q)$, $(M,\textit{g})$, and to the action by left translation in (*G*, *Q*), respectively. Note that $\overline{w}_Z^t= w_Z+t^{-1}\overline{w}_Z$.

On the one hand, $\mathcal {L}_{\pi '}\overline{X}$, the horizontal lift of $C_t^{-1}\overline{X}$ with respect to $\pi ' :M\times G\rightarrow M$ is given by

$$\begin{aligned} \mathcal {L}_{\pi '}C_t^{-1}\overline{X} = (\overline{X}, -tPU). \end{aligned}$$

On the other hand $d\overline{w}_Z(PU,PV) = \frac{1}{2}Q([PU,PV],Z)$ and

$$\begin{aligned} d\overline{w}_Z^t(\mathcal {L}_{\pi '}C_t^{-1}\overline{X},\mathcal {L}_{\pi '}C_t^{-1}\overline{Y})&= d\overline{w}_Z^t((\overline{X},-tPU),(\overline{Y},-tPV)),\\&= dw_Z(\overline{X},\overline{Y}) + \frac{1}{t}d\overline{w}_Z(-tPU,-tPV),\\&= dw_Z(\overline{X},\overline{Y}) + \frac{t}{2}Q([PU,PV],Z). \end{aligned}$$

Finally, note that

$$\begin{aligned} (\textit{g}{+}\frac{1}{t}Q)(Z^*,Z^*) = \textit{g}(Z^*,Z^*) + \frac{1}{t}Q(Z,Z) = \textit{g}(Z^*,Z^*) + \frac{1}{t} = \frac{1}{t}\left( t\textit{g}(Z^*,Z^*) + 1\right) \end{aligned}$$

once $Q(Z,Z) = 1$. The proof is finished by applying Claim 2 to the metric $\textit{g}{+} \frac{1}{t}Q$. Equation (15) follows easily combining Eq. (10) with (13), (14). $\square$

## Cheeger deformations on associated fiber bundles

In this section we shall use Cheeger deformations to produce deformed metrics on fiber bundles with compact structure groups. As we will describe in Sect. 4, such a deformation works as a *canonical model* from which any *basic* vertical metric deformation on fiber bundles shall descend from.

Let $F\hookrightarrow M{\mathop {\rightarrow }\limits ^{\pi }}B$ be a fiber bundle from a manifold *M*, with fiber *F* and compact structure group *G* and base *B*. We start by recalling that the structure group of a fiber bundle is the group where some choice of transition functions on *M* takes values. Precisely, if *G* acts effectively on *F*, then *G* is a structure group for $\pi$ if there is a choice of local trivializations $\{(U_i,\phi _i:\pi ^{-1}(U_i) \rightarrow U_i\times F)\}$ such that, for every *i*, *j* with $U_i\cap U_j \ne \emptyset$, there is a continuous function $\varphi _{ij} : U_i\cap U_j \rightarrow G$ satisfying

16 $$\begin{aligned} \phi _i\circ \phi _j^{-1}(p,f) = (p,\varphi _{ij}(p)f), \end{aligned}$$

for all $$p\in U_i\cap U_j$$.

The existence of $\{\varphi _{ij}\}$ allows us to construct a principal *G*-bundle over *B* (see [14, Proposition 5.2] for details) that we shall denote by $\mathcal {P}$. Furthermore, there exists a principal *G*-bundle $\overline{\pi }: \mathcal P\times F \rightarrow M$ whose principal action is given by

17 $$\begin{aligned} r (p,f) := (rp, rf). \end{aligned}$$

(For the details see the construction on the proof of [10, Proposition 2.7.1].)

For each pair $\textit{g}$ and $\textit{g}_F$ of *G*-invariant metrics on $\mathcal P$ and *F*, respectively, there exists a metric *h* on *M* induced by $\overline{\pi }$. Denote by $\mathcal {M}$ the set of all metrics obtained in this way (for instance, if the *G*-action on *F* is transitive, then every metric on *M* such that the holonomy acts by isometries on each fiber belongs to $\mathcal {M}$). The set $\mathcal {M}$ is the set of admissible metrics for our deformation:

### Definition 1

(*The deformation*) Given $h \in \mathcal {M}$, consider $\textit{g}{+}\textit{g}_F$ a product metric on $\mathcal P\times F$ such that $\overline{\pi }:(\mathcal P\times F,\textit{g} + \textit{g}_F)\rightarrow (M,\textit{h})$ is a Riemannian submersion. We define $\textit{h}_t$ as the submersion metric induced by $\textit{g}_t + \textit{g}_F$, where $\textit{g}_t$ is the time *t* Cheeger deformation associated with $\textit{g}$.

As it can be seen, the deformation itself is well-defined for a broader class of metrics (for instance, $\mathcal P\times F$ could have any metric such that each slice $\{p\}\times F$ has a *G*-invariant metric). However, if the metric is not a product metric, the deformed curvature is harder to control and Theorem 3.1 ahead does not hold.

### Curvature formulae

With the aim of establishing the basic curvature formulae associated to the introduced deformation, we proceed with the following discussion.

Fix $(p,f) \in \mathcal {P}\times F$. Any $\overline{X}\in T_{(p,f)}(\mathcal P\times F)$ can be written as $\bar{X} = \left( {X + V^{ \vee } ,X_{F}  + W^{*} } \right)$, where *X* is orthogonal to the *G*-orbit on $\mathcal P$, $X_F$ is orthogonal to the *G*-orbit on *F* and, for $V,W\in \mathfrak {g}$, $V^{\vee }$ and $W^{*}$ are the action vectors relative to the *G*-actions on $\mathcal P$ and *F* respectively. Let $P,~P_F$ and $P_t$ be the orbit tensors associated to $\textit{g},~\textit{g}_F$ and $\textit{g}_t$, respectively. We claim that $\overline{X}$ is $\textit{g}_t + \textit{g}_F$-orthogonal to the *G*-orbit of (17) if and only if

18 $$\begin{aligned} \overline{X} = (X-(P^{-1}_tP_FW)^{\vee },X_F + W^{*}). \end{aligned}$$

for some $$W\in \mathfrak {m}_f$$.

Indeed, a vector $(X+{V^{\vee }},X_F+W^{*})$ is horizontal if and only if, for every $U\in \mathfrak {g}$:

$$\begin{aligned} 0&= \left( \textit{g}_t{+}\textit{g}_F\right) ((X+V^{\vee },X_F+W^{*}), (U^{\vee },U^{*}))= \textit{g}(V^{\vee },U^{\vee }) + \textit{g}_F(W^{*},U^{*}) \\&= Q(P_tV + P_FW,U). \end{aligned}$$

Since *U* is arbitrary, we conclude that $V = -P^{-1}_tP_F W$.

Keeping in mind that the point (*p*, *f*) is fixed, we abuse notation and denote

19 $$\begin{aligned} d{\bar{\pi }}_{(p,f)}(X,X_F+U^*) := X+X_F+ U^*. \end{aligned}$$

Define the tensors ${\tilde{P}}_t,{\tilde{C}}_t:\mathfrak {m}_f\rightarrow \mathfrak {m}_f$,

20 $$\begin{aligned} {\tilde{P}}_t&:= P_F(1+P_t^{-1}P_F)^{-1}=(P_F^{-1}+P_t^{-1})^{-1}, \end{aligned}$$

21 $$\begin{aligned} {\tilde{C}}_t&:= -C_tP_t^{-1}{\tilde{P}}_t=-P^{-1}{\tilde{P}}_t. \end{aligned}$$

#### Claim 3

Let $\mathcal L_{\overline{\pi }}: T_{\overline{\pi }(p,f)}M \rightarrow T_{(p,f)}(\mathcal {P}\times F)$ be the horizontal lift associated to $\overline{\pi }$. Then,

22 $$\begin{aligned} \mathcal L_{\overline{\pi }}(X+X_F+U^*)=(X-(P_t^{-1}{\tilde{P}}_tU)^\vee ,X_F+(P_F^{-1}{\tilde{P}}_tU)^*). \end{aligned}$$

#### Proof

First observe that the right-hand-side of (22) is of the form (18): take $W=P_F^{-1} {\tilde{P}}_tU$, so $P_t^{-1}{\tilde{P}}_tU=P^{-1}_tP_FW$. Therefore, it is sufficient to verify that

$$\begin{aligned} d{\bar{\pi }} (X-(P_t^{-1}{\tilde{P}}_tU)^\vee ,X_F+(P_F^{-1}{\tilde{P}}_tU)^*)=X+X_F+U^*. \end{aligned}$$

Since $\ker d{\bar{\pi }}=\{(U^\vee ,U^*)~|~U\in \mathfrak {g}\}$, convention (19) gives $d{\bar{\pi }}(U^\vee ,0)=-U^*$, thus

$$\begin{aligned} d{\bar{\pi }} (X-(P_t^{-1}{\tilde{P}}_tU)^\vee ,X_F+(P_F^{-1}{\tilde{P}}_tU)^*)=X+X_F+((P_t^{-1}+P_F^{-1}){\tilde{P}}_tU)^*=X+X_F+U^* \end{aligned}$$

since $${\tilde{P}}_t=(P_t^{-1}+P_F^{-1})^{-1}$$. $$\square $$

Next, we prove that the unreduced sectional curvature is nondecreasing for reparameterized planes (Theorem 3.1). For the reparametrization, extend ${\tilde{C}}_t$ to $T_{\overline{\pi }(p,f)}M$ via

23 $$\begin{aligned} {\tilde{C}}_t(X+X_F + U^{*}) := X + X_F + ({\tilde{C}}_tU)^{*}. \end{aligned}$$

Mimicking M. Müter’s approach, we obtain a similar result to Theorem 2.2 defining ${\tilde{\kappa }}_{t}({\tilde{X}}, {\tilde{Y}}) = R_{\textit{h}_t}({\tilde{C}}_t^{-1}X,{\tilde{C}}_t^{-1}Y,{\tilde{C}}_t^{-1}Y,{\tilde{C}}_t^{-1}X)$.

#### Theorem 3.1

(Sectional curvature) Let $\textit{h}\in \mathcal M$ and $\textit{g}_t + \textit{g}_F$ be as in Definition 1. Then, for every pair ${\tilde{X}} = X + X_F + U^*$, ${\tilde{Y}} = Y + Y_F + V^*$ ,

$$\begin{aligned} {\tilde{\kappa }}_{t}({\tilde{X}}, {\tilde{Y}}) = \kappa _t(X{+}U^{\vee },Y{+}V^{\vee }) {+} K_{\textit{g}_F}(X_F - (P_F^{-1}PU)^*, Y_F - (P_F^{-1}PV)^*) + {\tilde{z}}_t({\tilde{X}},{\tilde{Y}}), \end{aligned}$$

where $\kappa _t$ is as in Theorem 2.2 and ${\tilde{z}}_t$ is non-negative.

#### Proof

The proof follows from a direct use of Gray–O’Neill curvature formula and Claim 3. Observe that

$$\begin{aligned} \mathcal {L}_{\overline{\pi }}({\tilde{C}}_t^{-1}{\tilde{X}}) = (C_t^{-1}(X+U^{\vee }),X_F - (P_F^{-1}PU)^{*}). \end{aligned}$$

Let ${\tilde{z}}_t$ be three times the norm squared of the integrability tensor of $\overline{\pi }$ applied to $\mathcal {L}_{\overline{\pi }}{\tilde{C}}_t^{-1}{\tilde{X}},\mathcal {L}_{\overline{\pi }}{\tilde{C}}_t^{-1}{\tilde{Y}}$ (see [10] for details). Then,

$$\begin{aligned}&R_{\textit{h}_t}({\tilde{C}}_t^{-1}X,{\tilde{C}}_t^{-1}Y,{\tilde{C}}_t^{-1}Y,{\tilde{C}}_t^{-1}X)=K_{\textit{g}_t}(C_t^{-1}(X+U^{\vee }),C_t^{-1}(Y+V^{\vee }))\\&\qquad +K_{\textit{g}_F}(X_F - (P_F^{-1}PU)^{*},Y_F - (P_F^{-1}PV)^{*})+{\tilde{z}}_t({\tilde{X}},{\tilde{Y}})\\&\quad =\kappa _t(X{+}U^{\vee },Y{+}V^{\vee }) {+} K_{\textit{g}_F}(X_F - (P_F^{-1}PU)^*, Y_F - (P_F^{-1}PV)^*) + {\tilde{z}}_t({\tilde{X}},{\tilde{Y}}). \end{aligned}$$

$$\square $$

Since the ${\tilde{z}}_t$ shall play some role in the next applications in this paper, we shall study it in more detail. We first claim that it isnon-decreasing with respect to *t*. This is a crucial observation since ${\tilde{z}}_0$ is an essential part of the initial curvature: since ${\bar{\pi }}:(\mathcal P\times F,g\times g_F)\rightarrow (M,h )$ is chosen to be a Riemannian submersion, ${\tilde{z}}_0$ is the *A*-tensor term on the submersion formula. Or, equivalently, taking $t=0$ in Theorem 3.1,

$$\begin{aligned}&K_{\textit{h}}({\tilde{X}},{\tilde{Y}})={\tilde{\kappa }}_{0}({\tilde{X}}, {\tilde{Y}}) \\&\quad\quad\quad\quad\; = \kappa _0(X{+}U^{\vee },Y{+}V^{\vee }) {+} K_{\textit{g}_F}(X_F - (P_F^{-1}PU)^*, Y_F - (P_F^{-1}PV)^*) + {\tilde{z}}_0({\tilde{X}},{\tilde{Y}})\\&\quad\quad\quad\quad\; = K_{\textit{g}}(X{+}U^{\vee },Y{+}V^{\vee }) {+} K_{\textit{g}_F}(X_F - (P_F^{-1}PU)^*, Y_F - (P_F^{-1}PV)^*) + {\tilde{z}}_0({\tilde{X}},{\tilde{Y}}). \end{aligned}$$

It is even possible to furnish a precise description to $\widetilde{z}_t$. Indeed, although we choose to omit the proof, it can be proved exactly as in [6, Lemma 3.5, p. 11], or Lemma 2, that

#### Lemma 3

Let

$$\begin{aligned} w^t_Z&: T\mathcal P \rightarrow \mathbb {R} \\&X+U^{\vee } \mapsto \textstyle \frac{1}{2}\textit{g}_t(X+ U^{\vee },Z^{\vee }). \\ w_Z&: TF \rightarrow \mathbb {R} \\&X_F + U^{*} \mapsto \textstyle \frac{1}{2}\textit{g}_F(X_F + U^{*},Z^*), \end{aligned}$$

where $Z^*$ is the action vector associated to $Z\in \mathfrak {g}$. Then it holds that

$$\begin{aligned}&3^{-1}{\tilde{z}}_t({\tilde{X}}, {\tilde{Y}}) = \\& \max _{Z\in \mathfrak {g}\setminus \{0\}}\dfrac{\left\{ dw_Z^t(X+C_t^{-1}U^{\vee },Y+C_t^{-1}V^{\vee })+dw_Z(X_F-(P_F^{-1}PU)^*,Y_F-(P_F^{-1}PV)^*)\right\} ^2}{\textit{g}_t(Z^{\vee },Z^{\vee })+\textit{g}_F(Z^*,Z^*)}. \end{aligned}$$

### Regularization via Cheeger deformations

Let $(M,\textit{g})$ be a compact Riemannian manifold with an effective isometric action by a compact Lie group *G*. If $\textit{g}_t$ denotes a one-parameter family of metrics obtained out from $\textit{g}$ via Cheeger deformations, we can check from the expression to $P_t = P(1+tP)^{-1}$, see Eq. (1), that as $t\rightarrow \infty$ the Riemannian metric $\textit{g}_t$ degenerates.

Taking this observation in account, in [18] Searle, Solórzano and Wilhelm show that for any compact subset of the regular stratum of the *G*-action on *M*, re-scaling the fibers of the fiber bundle $\pi : (M^{reg},\textit{g}_t) \rightarrow M^{reg}/G$, with the same parameter *t*, a procedure known as *Canonical Variation*, implies that for any integer $p\ge 0$ it holds the convergence of ${\widetilde{g}}_t: = t{\textit{g}_t}|_\mathcal{V}\oplus \textit{g}|_\mathcal{H}$, in the $C^p$-topology, to a Riemannian metric with totally geodesic fibers, see [18, Theorem A].

It follows, in particular, that the class $\mathbf {P}^{\Omega }$ of principal bundles with *connection metrics* is invariant by Cheeger deformations. More interesting, any principal bundle with an invariant submersion metric is attracted to $\mathbf {P}^{\Omega }$ by the means of Cheeger deformations.

In this section, we shall prove the analogous result to the class of Riemannian submersions on fiber bundles with compact structure group and total space.

Let $(M,\textit{h}_t)\rightarrow (B,\textit{g}_B)$ be a complete Riemannian fiber bundle where $\textit{h}_t$ is induced via Definition 1. We prove that for any compact $K\subset M$ and any non-negative integer *p*, the canonical deformation

24 $$\begin{aligned} \widetilde{\textit{h}_{t}}(x) := t\textit{h}_t(x)|_\mathcal{V} + \textit{h}_t(x)|_\mathcal{H},~x\in K \end{aligned}$$

where $(\mathcal H,\textit{h}_t|_\mathcal{H})$ is isometric to $(TB,\textit{g}_B)$, converges to, in the $C^p$-topology, to a Riemannian submersion metric with totally geodesic fibers on $M\rightarrow B$ and horizontal distribution isometric to $\mathcal H\cong TB$.

To begin with, let us explain a little bit further about the admissible metrics to the deformation since we have the restriction imposed by the class $\mathcal M$ (recall Definition 1).

Suppose that we start with a Riemannian metric $\textit{g}_B$ on *B*. Then let $\mathcal P$ be the principal bundle obtained from $M\rightarrow B$ and let $\omega : TP \rightarrow \mathfrak {g}$ be any connection there defined. If the structure group of $M\rightarrow B$ has a biinvariant metric *Q*, we impose the *Kaluza–Klein (or connection) metric* on $\mathcal P$:

25 $$\begin{aligned} \textit{g} := \textit{g}_B + Q(\omega ,\omega ). \end{aligned}$$

Assuming that *G* is compact one observes that it is always possible to assume that $\omega$ is *G*-invariant. Therefore, any metric $\textit{h}$ obtained this way belongs to $\mathcal M$ for the invariant metrics on $\mathcal P$ defined by Eq. (25). Theorem 3.2 shall show that every metric *h* in the class $\mathcal M$ is attracted by the subset of $\mathcal M$ of metrics obtained in this manner.

Let us first prove that the metric (24) shall approach a metric with totally geodesic fibers as *t* grows large. To do so, we first observe that the shape operator ${\widetilde{S}}_X$ for any $X\in \mathcal H$ associated to $\widetilde{\textit{h}_t}$ coincides with the shape operator $S^t_X$ of $\textit{h}_t$. Indeed, according to equation (2.1.7) in [10, Chapter 2, p. 47] the *vertical component* of the covariant derivative ${\widetilde{\nabla }}_TX$ of a canonical variation remains unchanged provided if *T* is vertical and *X* is horizontal.

It then suffices to check that $\lim _{t\rightarrow \infty }\max _{|X| = 1}|S^t_X|_t = 0$ in *K*. To do so, observe that since the shape operator is symmetric with respect to the metric it is obtained from, it holds that

$$\begin{aligned} \max _{|X|_{\textit{h}}=1}|S^t_X|_{\textit{h}_t} = \max _{|X|_{\textit{h}}= |V|_{\textit{h}}=1}|\textit{h}(-\widetilde{C}_t{{\nabla _t}^{\mathbf {v}}_VX},V)|. \end{aligned}$$

Finally, it can be directly checked from the Koszul formula that the right hand side on the above equation goes to 0 as $t\rightarrow \infty$: since

$$\begin{aligned} \textit{h}(-{\widetilde{C}}_t{{\nabla _t}^{\mathbf {v}}_VX},V)&= X\textit{h}({\widetilde{C}}_tV,V) + \textit{h}([X,V],{\widetilde{C}}_tV) + \textit{h}([X,V],{\widetilde{C}}_tV) \end{aligned}$$

and ${\widetilde{C}}_t = -P^{-1}(P_F^{-1} + P_t^{-1})^{-1}$, $P_t^{-1} = P^{-1}(1+tP)$ it is clear that the only possible problematic term is $X\textit{h}({\widetilde{C}}_tV,V)$. However, applying the Leibiniz rule, one gets that

$$\begin{aligned} X\textit{h}({\widetilde{C}}_tV,V) = \textit{h}(\nabla _X{\widetilde{C}}_tV,V) + \textit{h}({\widetilde{C}}_tV,\nabla _XV). \end{aligned}$$

Once more, the Leibiniz rule and the notion of covariant derivative to tensors implies that it suffices to study $\nabla _X{\widetilde{C}}_t$. But since

$$\begin{aligned} \nabla _X{\widetilde{C}}_t&= -(P^{-2}\nabla _XP)(P_F^{-1} + P_t^{-1})^{-1} + P^{-1}\nabla _X(P_F^{-1} + P_t^{-1})^{-1},\\ \nabla _X(P_F^{-1} + P_t^{-1})^{-1}&= -(P_F^{-1} + P_t^{-1})^{-2}(\nabla _XP_F^{-1} -P_t^{-2}\nabla _XP_t),\\ \nabla _XP_t&= \nabla _X(1+tP) = t\nabla _XP. \end{aligned}$$

we are done. $\square$

We prove that:

#### Theorem 3.2

Let $\pi : F\hookrightarrow M \rightarrow B$ be a fiber bundle with compact total space and compact structure group *G*. Assume that $\textit{h}$ is a Riemannian submersion metric on *M* obtained via the submersion $\overline{\pi }: (\mathcal  P\times F,\textit{g}+\textit{g}_F) \rightarrow M$, where $\mathcal P$ is the associated principal bundle to $\pi$ and $\textit{g}, \textit{g}_F$ are, respectively, *G*-invariant metrics on $\mathcal P$ and *F*. Then for any integer $p\ge 0$, after an appropriate re-scaling the fibers of $\pi$, the metric deformation $\textit{h}_t$ (Definition 1, Sect. 3), converges in the $C^p$-topology to a Riemannian submersion metric with totally geodesic fibers.

#### Proof

Although we could proceed similarly but independently to Searle–Solórzano–Wilhelm’s result ([18, Theorem A]), we shall use it to both metrics $\textit{g}$ in $\mathcal P$ and $\textit{g}_F$ in *F*.

Once more, Theorem A in [18] states that if the metric $\widetilde{\textit{g}}_t$ is a same parameter canonical variation of the Cheeger deformation of $\textit{g}$, it follows that for any integer $p\ge 0$ the metric $\widetilde{\textit{g}}_t$ converges, in the $C^p$ topology, to a metric $\textit{g}_{\infty }$ to which the fibers of $\pi$ are totally geodesic. It then suffices to check that a canonical variation of $\textit{g}_t$ and of the *t*-Cheeger deformation of $\textit{g}_F$ induces via $\overline{\pi }: (\mathcal P + F,\textit{g}_t + \textit{g}_F) \rightarrow (M,\textit{h}_t)$ the metric $\widetilde{h}_t$.

To do so, observe that since according to Eq. (22)

$$\begin{aligned} \mathcal {L}_{\overline{\pi }}(X+X_F+U^*)=(X-(P_t^{-1}{\tilde{P}}_tU)^\vee ,X_F+(P_F^{-1}{\tilde{P}}_tU)^*) \end{aligned}$$

one has

$$\begin{aligned} \textit{h}_t\left( d\overline{\pi }\mathcal {L}_{\overline{\pi }}(X+X_F+U^*),\cdot \right)&= \textit{g}_t + \textit{g}_F\left( (X-(P_t^{-1}{\tilde{P}}_tU)^\vee ,X_F+(P_F^{-1}{\tilde{P}}_tU)^*),\cdot \right) \\&= \textit{g}_t\left( X-(P_t^{-1}{\tilde{P}}_tU)^\vee ,\cdot \right) + \textit{g}_F\left( X_F+(P_F^{-1}{\tilde{P}}_tU)^*,\cdot \right) \\&= \textit{g}(X,\cdot ) + \textit{g}(-C_t(P_t^{-1}{\tilde{P}}_tU)^{\vee },\cdot ) + \textit{g}_F\left( X_F+(P_F^{-1}{\tilde{P}}_tU)^*,\cdot \right) . \end{aligned}$$

The tensor $P_t$ is changed to $tP(1+tP)^{-1}$ under a canonical variation so the following changes hold

$$\begin{aligned} C_t&= P^{-1}P_t \leftrightarrow t(1+tP)^{-1}\\ {\tilde{P}}_t&= \left(P_F^{-1} + P_t^{-1}\right)^{-1} \leftrightarrow \left(P_F^{-1} + t^{-1}P_t^{-1}\right)^{-1}. \end{aligned}$$

Moreover, the *t*-canonical variation of a *t*-Cheeger deformation of $\textit{g}_F$ yields the change

$$\begin{aligned} P_F \leftrightarrow tP_F(1+tP_F)^{-1} \end{aligned}$$

Hence, making $t\rightarrow \infty$ yields

$$\begin{gathered}   g\left( { - \left( {P^{{ - 1}} \left( {t^{{ - 1}} P_{F}^{{ - 1}} (1 + tP_{F} ) + t^{{ - 1}} P_{t}^{{ - 1}} } \right)^{{ - 1}} U} \right)^{ \vee } , \cdot } \right) \hfill \\   \quad  + g_{F} \left( {\left( {t^{{ - 1}} P_{F}^{{ - 1}} (1 + tP_{F} )\left( {t^{{ - 1}} (1 + tP_{F} )P_{F}^{{ - 1}}  + t^{{ - 1}} P_{t}^{{ - 1}} } \right)^{{ - 1}} U} \right)^{*} , \cdot } \right) \hfill \\   \quad  \to \frac{1}{2}\left\{ {g_{F} (U^{*} , \cdot ) - g\left( {(P^{{ - 1}} U)^{ \vee } , \cdot } \right)} \right\} \hfill \\  \end{gathered}$$

On the other hand, since $t{\tilde{C}}_t \rightarrow P^{-1}$ as $t\rightarrow \infty$ and $d\overline{\pi }(U^{\vee },0) = -U^{*}$ (recall Claim 3), the previous computation guarantees the result. $\square$

## Revisiting some classical results: curvature of bi-quotients and almost non-negative curvatures

### Bi-quotients

Following [10, Chapter 2.6], let *G* be a Lie group with a left-invariant metric that is right-invariant under a subgroup *H*. Inspired by constructions of homogeneous spaces, consider the group manifold $G\times H$ which acts isometrically on *G* via

26 $$\begin{aligned} (g,h)\cdot a := gah^{-1}. \end{aligned}$$

Since any subgroup $K\le G\times H$ acts via the same manner on *G*, if this *K*-action happens to be free then the the orbit space *G*//*K* is called a *bi-quotient* of *G*. Moreover, once the *K* action on *G* is via isometries, there is a metric on *G*//*K* that makes the quotient projection $\pi : G\rightarrow G//K$ to be a Riemannian submersion.

Observe that we can see *G*//*K* as the total space of a fiber bundle with trivial fiber, in the sense that the fiber *F* is a just point and the bundle projection is the identity map. This manner, we can derive the curvature formula to certain deformed metrics on bi-quotients from the previous section.

Indeed, note that if we consider $G\times K$ with the *K*-action defined by (4) then the projection $\pi '$ recovers *G* as the respective orbit space. Therefore, composing $\pi '$ with the projection $\pi$ we obtain the corresponding projection $\overline{\pi }$ (recall Definition 1)27The purpose of the computations presented here is the one of showing how the curvature formula on bi-quotients can be very simplified considering the deformation we have introduced in Sect. 3.

Indeed, a simple use of Theorem 3.1 implies that the sectional curvature of $M := G//H$ can be read from $\overline{\pi }$ via the curvature of $\mathcal P = G$ since $F = \{e\},$ the identity in *K*. Namely,

#### Theorem 4.1

Let *Q* be a bi-invariant metric on $K = G\times H$ and $\textit{g}$ be a left invariant metric on *G* which is right-invariant by the elements of the form $\{e\}\times H$. If $\textit{g}_t$ is the metric on *G*//*K* induced by $\overline{\pi }: (G\times K,\textit{g} + t^{-1}Q) \rightarrow (G//K,\textit{h}_t)$, the sectional curvature of $\textit{h}_t$ satisfies

28 $$\begin{aligned} \kappa _{\textit{h}_t}\left({\tilde{X}}, {\tilde{Y}}\right) = \kappa _{\textit{g}_t}\left(X{+}U^{\vee },Y{+}V^{\vee }\right) + \widetilde{z} _t \left({\tilde{X}},{\tilde{Y}}\right) \end{aligned}$$

where ${\tilde{X}} = X + X_F + U^*$, ${\tilde{Y}} = Y + Y_F + V^*$ and ${\tilde{z}}_t$ is computed in Lemma 3.

We proceed developing the formulae to the Ricci curvature of the metric deformation given in Definition 1, presented in Sect. 3. Further applications on bi-quotients shall be also obtained next.

### Almost non-negative curvatures and positive Ricci curvature

Since we are relying on the horizontal lift $\mathcal L_{\overline{\pi }}$ defined by (22) to compute curvatures, in order to study the Ricci curvature of $\textit{h}_t$ we begin by constructing an appropriate basis for the horizontal space of $\overline{\pi }$ with respect to $\textit{g}_t + \textit{g}_F.$

Consider a *Q*-orthonormal basis $\{v_k(0)\}$ of $\mathfrak {m}_f$ and define

$$\begin{aligned} v_k(t) = {\tilde{P}}_t^{-1/2}v_k(0). \end{aligned}$$

#### Lemma 4

The set

29 $$\begin{aligned} \{(-{P_t^{-1}{\tilde{P}}_tv_k(t)}^{\vee },{P_F^{-1}{\tilde{P}}_tv_k(t)}^*)\} = \{(-P_t^{-1}{\tilde{P}}_t^{1/2}v_k(0)^\vee , P_F^{-1}{\tilde{P}}_t^{1/2}v_k(0)^*)\} \end{aligned}$$

is $\textit{g}_t + \textit{g}_F$-orthonormal and $\textit{g}_t + \textit{g}_F$ orthogonal to $(U^\vee ,U^*)$, for every $U\in \mathfrak {g}$.

#### Proof

Note that the elements in (29) are of the form (22). Thus, it is sufficient to show that the set (29) is orthonormal. A straightforward computation gives:

$$\begin{gathered}   \left( {g_{t}  + g_{F} } \right)\left( {\left( { - P_{t}^{{ - 1}} \tilde{P}_{t} v_{i} (t)^{ \vee } ,P_{F}^{{ - 1}} \tilde{P}_{t} v_{i} (t)^{*} } \right),\left( { - P_{t}^{{ - 1}} \tilde{P}_{t} v_{j} (t)^{ \vee } ,P_{F}^{{ - 1}} \tilde{P}_{t} v_{j} (t)^{*} } \right)} \right) \hfill \\   \quad  = Q\left( {\tilde{P}_{t} v_{i} (t),P_{t}^{{ - 1}} \tilde{P}_{t} v_{j} (t)} \right) + Q\left( {\tilde{P}_{t} v_{i} (t),P_{F}^{{ - 1}} \tilde{P}_{t} v_{j} (t)} \right) \hfill \\   \quad  = Q\left( {\tilde{P}_{t} v_{i} (t),\left( {P_{t}^{{ - 1}}  + P_{F}^{{ - 1}} } \right)\tilde{P}_{t} v_{j} (t)} \right) \hfill \\   \quad  = Q\left( {\tilde{P}_{t} v_{i} (t),v_{j} (t)} \right) = Q\left( {\tilde{P}_{t}^{{1/2}} v_{i} (0),\tilde{P}_{t}^{{ - 1/2}} v_{j} (0)} \right) = \delta _{{ij}} , \hfill \\  \end{gathered}$$

where we have used that $(P_t^{-1} + P_F^{-1}) = {\tilde{P}}_t^{-1}$ and that ${\tilde{P}}_t$ is symmetric. $\square$

Let $\{e_i^{B}\}$ and $\{e_j^F\}$ be orthonormal bases for the spaces normal to the orbits on $\mathcal P$ and on *F*, respectively. We complete the set on Lemma 4 to a $\textit{g}_t + \textit{g}_F$-othornormal basis for the $\bar{\pi }$-horizontal space:

30 $$\begin{aligned} \mathcal {B}_t := \left\{ \left(e_i^B,0 \right), \left(-{P_t^{-1}{\tilde{P}}_t^{1/2}v_k(0)}^{\vee },{P_F^{-1}{\tilde{P}}_t^{1/2}v_k(0)}^*\right), \left(0,e_j^F \right)\right\} . \end{aligned}$$

Denote by $e_1,\ldots ,e_n$ the elements in $\mathcal B_t$.

#### Lemma 5

For any $(p,f)\in \mathcal P\times F$ and $X+X_F+U^*\in T_{\bar{\pi }(p,f)}M$,

31 $$\begin{aligned} \lim _{t\rightarrow \infty }{\mathrm{Ric}}_{\textit{h}_t}(X + X_F + U^*) \ge {\mathrm{Ric}}_{\textit{g}}^{\mathbf {h}}(X) + {\mathrm{Ric}}_{\textit{g}_F}^{\mathbf {h}}(X_F) + \sum _k\frac{1}{4}\Vert [v_k(0),U]\Vert _Q^2. \end{aligned}$$

#### Proof

Using the basis $\mathcal B_t$, from (30), and Theorem 3.1, we have:

$$\begin{aligned}&{\mathrm{Ric}}_{\textit{h}_t}({\tilde{X}})=\sum _{i=1}^n\kappa _t({\tilde{C}}_t{\tilde{X}},{\tilde{C}}_t e_i)\\& \quad\quad\quad\;\; \ge\sum _i \kappa _t(X-(C_tP_t^{-1}{\tilde{P}}_tU)^{\vee },e_i^B) + \sum _{k}\kappa _t(X - (C_tP_t^{-1}{\tilde{P}}_tU)^{\vee },-C_tP_t^{-1}{\tilde{P}}_t^{1/2}v_k(0)^{\vee })\\&\quad\quad\quad\quad\quad + \sum _{j}K_{\textit{g}_F}(X_F + (P_F^{-1}{\tilde{P}}_tU)^*,e^F_j) + \sum _{k}K_{\textit{g}_F}(X_F+(P_F^{-1}{\tilde{P}}_tU)^*,P_F^{-1}{\tilde{P}}_t^{1/2}v_k(0)^*). \end{aligned}$$

On the other hand, the ${\tilde{P}}_t$ satisfies:

32 $$\begin{aligned} \lim _{t\rightarrow \infty }t{\tilde{P}}_t&= 1, \end{aligned}$$

33 $$\begin{aligned} \underset{t\rightarrow \infty }{\lim }P_t^{-1}{\tilde{P}}_t&= 1. \end{aligned}$$

In particular, ${\tilde{P}}_t \rightarrow 0$ as $t\rightarrow \infty$. Equation (32) follows since $t{\tilde{P}}_t = P_F(P_t+P_F)^{-1}tP_t$, $P_t \rightarrow 0$ and $tP_t \rightarrow 1$. Equation (33) follows since

$$\begin{aligned} \lim _{t\rightarrow \infty }P_t^{-1}{\tilde{P}}_t=\lim _{t\rightarrow \infty }(tP_t)^{-1}\lim _{t\rightarrow \infty }t{\tilde{P}}_t=1. \end{aligned}$$

Using (32), we observe that

34 $$\begin{aligned}&\lim _{t\rightarrow \infty }\left\{ \sum _{j}K_{\textit{g}_F}\left(X_F + (P_F^{-1}{\tilde{P}}_tU)^*,e^F_j \right) + \sum _{k}K_{\textit{g}_F}(X_F+(P_F^{-1}{\tilde{P}}_tU)^*,P_F^{-1}{\tilde{P}}_t^{1/2}v_k(0)^*)\right\} \nonumber \\&\quad\quad\;={\mathrm{Ric}}_{\textit{g}_F}^{\mathbf{h}}(X_F). \end{aligned}$$

Moreover, using Eq. (5) and that $C_tP_t^{-1} = P^{-1}$,

35 $$\begin{aligned} \lim _{t\rightarrow \infty }\sum _i \kappa _t(X-(C_tP_t^{-1}{\tilde{P}}_tU)^{\vee },e_i) \ge \lim _{t\rightarrow \infty }{\mathrm{Ric}}_{\textit{g}}^{\mathbf{h}}(X-(P^{-1}{\tilde{P}}_tU)^{\vee })={\mathrm{Ric}}_{\textit{g}}^{\mathbf{h}}(X). \end{aligned}$$

For the remaining term:

$$\begin{aligned}&\kappa _t(C_tX - (C_tP_t^{-1}{\tilde{P}}_tU)^{\vee },-C_tP_t^{-1}{\tilde{P}}_t^{1/2}v_k(0)^{\vee }) \\&\quad \ge K_{\textit{g}}(X-(P^{-1}{\tilde{P}}_tU)^{\vee },-P^{-1}{\tilde{P}}_t^{1/2}v_k(0)^{\vee }) + \frac{t^3}{4}\Vert [{\tilde{P}}_tU,{\tilde{P}}_t^{1/2}v_k(0)]\Vert ^2_Q \end{aligned}$$

Using (32) and (33), we obtain

36 $$\begin{aligned} \lim _{t\rightarrow \infty }\kappa _t(C_tX - (C_tP_t^{-1}{\tilde{P}}_tU)^{\vee },-C_tP_t^{-1}{\tilde{P}}_t^{1/2}v_k(0)^{\vee }) \ge \frac{1}{4}\Vert [v_k(0),U]\Vert _Q^2. \end{aligned}$$

Lemma 5 follows by putting together (34), (35) and (36). $\square$

The following needed lemmas can be readily obtained via straightforward computations, therefore, we chose to not include their proofs.

#### Lemma 6

It holds that

37 $$\begin{aligned} \lim _{t\rightarrow \infty }{\tilde{z}}_t({\tilde{C}}_t{\tilde{X}},{\tilde{C}}_t{\tilde{Y}}) = 3\max _{Z\in \mathfrak {g}\setminus \{0\}}\dfrac{\lim _{t\rightarrow \infty }\left\{ dw^t_Z(X-U^{\vee },Y-V^{\vee }) + dw_Z(X_F,Y_F)\right\} ^2}{\textit{g}_F(Z^*,Z^*)}. \end{aligned}$$

Moreover, if the orbits on $\mathcal P$ are totally geodesic then

38 $$\begin{aligned} \lim _{t\rightarrow \infty }dw^t_Z(X-U^{\vee },Y)^2 = \textit{g}(A_XU,Z)^2. \end{aligned}$$

#### Lemma 7

If $\{e_i\}$ denotes the set of elements in the basis $\mathcal B_t$ (see Eq. 30) and the *G* orbits on $\mathcal P$ are totally geodesic then for any $\widetilde{X} = X + X_F + U^*$ it holds that

39 $$\begin{aligned} \lim _{t\rightarrow \infty }\sum _i{\tilde{z}}_t({\tilde{C}}_t d\overline{\pi }e_i, {\tilde{C}}_t{\tilde{X}}) = 3\sum _{i=1}|A^{\pi }_Xe^B_i|^2_{\textit{g}} + 3\sum _{j=1}|A^{\pi _F}_{X_F}e_j^F|_{g_F}^2. \end{aligned}$$

Therefore,

40 $$\begin{aligned} \lim _{t\rightarrow \infty }{\mathrm{Ric}}_{\textit{h}_t}(X + X_F + U^*)\;=\;&{\mathrm{Ric}}_{\bar{\textit{g}}}(d\pi X) + {\mathrm{Ric}}^{\mathbf {h}}(X_F) + 3\sum _{j=1}|A^{\pi _F}_{X_F}e_j^F|_{g_F}^2 \hfill \\ & + \sum _k\frac{1}{4}\Vert [v_k(0),U]\Vert _Q^2. \end{aligned}$$

We now recover the results of Schwachhöfer and Tuschmann ([19]) relating the geometry and the topology of bi-quotients. The method applied provides a huge simplification over their work. We re-inforce, however, that the possibility of a simplification was already expected, as observed Wilking and Ziller, see [25]. More precisely, it is always possible by the means of a Cheeger deformation to prove the existence of metrics of almost non-negative sectional curvature on cohomogeneity one manifolds: the idea consists of putting metrics on non-negative sectional curvature near the singular orbits (recall for instance that these are homogeneous disk-bundles ([11])) and extend these arbitrarily in the middle. Then it can be shown that all curvatures in the middle go to 0.

In what follows the method is very different, but it relies in the same spirit: Cheeger deformations tend to shrink the curvature along the orbits. More importantly, they also work as a regularization process: the metrics naturally converge to a metric with totally geodesic fibers. This phenomenon is manifested here in the form that the Ricci curvature of a bi-quotient is completely determined by the Ricci curvature of the upstairs Lie group with a bi-invariant metric.

#### Theorem 4.2

(Schwachhöfer–Tuschmann) A bi-quotient *G*//*K* of a compact connected Lie group *G* carries a metric of positive Ricci curvature if, and only if, its fundamental group is finite.

#### Proof

Recall that according to Sect. 4.1, *G*//*K* can be seen as the total space with trivial fiber $F = \{e\}$ and structure group *K*. Since the tangent space to the ‘manifold point’ $\{e\}$ is only the zero vector it follows that ${\mathrm{Ric}}_{\textit{g}_F}^{\mathbf{h}} \equiv 0$. Moreover, denote by $\pi : K\hookrightarrow G\rightarrow G//K$ the Riemannian submersion obtained from the principal bundle with total space $\mathcal P = G$. According to Lemma 7, if $\overline{\textit{g}}$ denotes the submersion metric induced by $\pi$ from $\textit{g}$ one gets

$$\begin{aligned} \lim _{t\rightarrow \infty }{\mathrm{Ric}}_{\textit{h}_t}(X + X_F + U^*)&= {\mathrm{Ric}}_{\bar{\textit{g}}}(d\pi X) + 3\sum _{j=1}|A^{\pi _F}_{X_F}e_j^F|_{g_F}^2 + \frac{1}{4}\sum _k\Vert [v_k(0),U]\Vert _Q^2\\&={\mathrm{Ric}}_{\bar{\textit{g}}}(d\pi X) + \frac{1}{4}\sum _k\Vert [v_k(0),U]\Vert _Q^2, \end{aligned}$$

where the last equality comes from the fact that the horizontal space associated to the action of *K* in $F = \{e\}$ is only the zero vector, i.e, $X_F = 0$. Now the proof is finished by noticing that if $|\pi _1(G//K)| < \infty$ then the same holds for *G*.

Therefore, since

$$\begin{aligned} {\mathrm{Ric}}_{\bar{\textit{g}}}(d\pi X)&= {\mathrm{Ric}}^{\mathbf{h}}(X) + 3\sum _{i=1}^{\dim G//K}|A^{\pi }_Xe_i|^2, \end{aligned}$$

where $\{e_1,\ldots ,e_{\dim G//K}\}$ is an orthonormal basis to the horizontal space of the *K*-action on *G*, associated to the submersion $\pi$, if *Q* is any bi-invariant metric on *G* it holds that

$$\begin{aligned} {\mathrm{Ric}}_{\bar{\textit{g}}}(d\pi X)&= {\mathrm{Ric}}^{\mathbf{h}}(X) + 3\sum _{i=1}^{\dim G//K}|A^{\pi }_Xe_i|^2,\\&= \sum _{i=1}^{\dim G//K}\Vert [X,e_i]\Vert ^2_Q + \tfrac{3}{4}\sum _{i=1}^{\dim G//K}\Vert [X,e_i]^{\mathfrak {k}}\Vert _Q^2, \end{aligned}$$

where $\mathfrak {k}$ is the Lie algebra of *K*. Hence,

$$\begin{aligned} \lim _{t\rightarrow \infty }{\mathrm{Ric}}_{\textit{h}_t}(X + X_F + U^*) & = \sum _{i=1}^{\dim G//K}\Vert [X,e_i]^{\mathfrak {m}}\Vert ^2_Q + \tfrac{7}{4}\sum _{i=1}^{\dim G//K}\Vert [X,e_i]^{\mathfrak {k}}\Vert _Q^2 \hfill \\ & \quad\quad + \frac{1}{4}\sum _k\Vert [v_k(0),U]\Vert _Q^2 \end{aligned}$$

and so $\lim _{t\rightarrow \infty }{\mathrm{Ric}}_{\textit{h}_t}(X + X_F + U^*) > 0$ since *G* has finite fundamental the sums of the three kind of brackets cannot vanish simultaneously. $\square$

We finish providing a result about almost non-negative sectional curvature and positive Ricci curvature, simultaneously, to biquotients. This shall be done taking advantage of the following similar result to [7, Theorem 0.18], which has a very simple proof. Once more, this proof was already known to be possible by B. Wilking and W. Ziller in the context of principal fiber bundles. Here we extent it naturally to general fiber bundles with compact structure group.

#### Theorem 4.3

(Fukaya–Yamaguchi type result) Let $F \hookrightarrow M \rightarrow B$ be a bundle with compact structure group *G*, fiber *F* and base *B*. Assume that *M* is an associate bundle to $\pi : (\mathcal P,\textit{g}) \rightarrow B$ such that:

1. $$K_{\textit{g}}\ge 0$$;
2. *F* has a *G*-invariant metric $\textit{g}_F$ of non-negative sectional curvature.

Then *M* admits a sequence of Riemannian metrics $\{\textit{g}_n\}$ such that $\mathrm {sec}_{\textit{g}_n} \ge -\frac{1}{n},$ $\mathrm {diam}~(M,\textit{g}_n) \le \frac{1}{n}.$

#### Definition 2

A compact manifold *M* with a family of metrics $(\textit{g}_n)$ as on the thesis of Theorem 4.3 is said to admit *almost non-negative sectional curvature*.

Theorem 4.3 is a straightforward consequence of the following lemma.

#### Lemma 8

Let $F\hookrightarrow M \rightarrow B$ and $\pi : \mathcal P \rightarrow B$ as on the hypotheses of Theorem 4.3. Then for each $\epsilon > 0$ there exists $t_{\epsilon } > 0$ such that for every $t > t_{\epsilon },$ ${\tilde{\kappa }}_t(\widetilde{X},\widetilde{Y}) \ge -\epsilon ,~|\widetilde{X}| = |\widetilde{Y}| = 1.$

#### Proof

Assume by contradiction that there is $\epsilon > 0$, a sequence $\{t_n\}\nearrow +\infty$ and a sequence of planes $\{\widetilde{X}_n,{\widetilde{Y}}_n\}$ with $|\widetilde{X}_n| = |\widetilde{Y}_n| = 1$ satisfying

41 $$\begin{aligned} {\tilde{\kappa }}_n \left({\widetilde{X}}_n,{\widetilde{Y}}_n \right) \le -\epsilon . \end{aligned}$$

By compactness, passing to a subsequence if necessary one extracts a limit plane $\{{\widetilde{X}},{\widetilde{Y}}\}$ such that

$$\begin{aligned} -\epsilon \ge \lim _{n\rightarrow \infty }\left\{ \kappa _n \left(X_n{+}U_n^{\vee },Y_n{+}V_n^{\vee }\right) {+} K_{\textit{g}_F}\left((X_F)_n - (P_F^{-1}PU_n)^*, (Y_F)_n - (P_F^{-1}PV_n)^*\right)\right\} . \end{aligned}$$

Theorem 2.2 then implies that

42 $$\begin{aligned} -\epsilon \ge \lim _{n\rightarrow \infty }\kappa _n \left(X_n{+}U_n^{\vee },Y_n{+}V_n^{\vee }\right) \ge \lim _{n\rightarrow \infty }\left\{ \kappa _0(X_n+U_n^{\vee },Y_n + V_n^{\vee } + n^3|[U_n,V_n]|_Q^2\right\} . \end{aligned}$$

and hence, $\lim _{n\rightarrow \infty }[U_n,V_n] = 0$. Therefore,

43 $$\begin{aligned} -\epsilon \ge \kappa _0 \left(X+U^{\vee },Y{+}V^{\vee }\right) {+} K_{\textit{g}_F}\left(X_F-(P_F^{-1}PU)^*,Y_F - (P_F^{-1}PV)^*\right) \ge 0. \end{aligned}$$

$$\square $$

We thus conclude:

#### Theorem 4.4

(Schwachhöfer–Tuschmann) Any bi-quotient *G*//*K* from a compact Lie group *G* admits a metric with positive Ricci curvature and almost non-negative sectional curvature simultaneously if, and only if, *G*//*K* has finite fundamental group.

#### Remark 1

Since $\mathcal P$ is a principal bundle, we could try to impose more rigid hypotheses to produce a metric of non-negative sectional curvature, but this is not possible only via the above method. Indeed, even assuming that $G = S^3, SO(3)$ and that $\textit{g}_F$ and $\textit{g}$ have positive sectional curvature we would have that

$$\begin{aligned} \kappa _0 \left(X,Y{+}V^{\vee }\right) {+} K_{\textit{g}_F}\left(X_F,Y_F - (P_F^{-1}PV)^*\right) = 0 \end{aligned}$$

for planes $X = 0,U = 0, V = 0, Y_F = 0.$ In this case,

$$\begin{aligned} \widetilde{X} = X_F,~\widetilde{Y} = Y, \end{aligned}$$

so we get no contradiction for any $\epsilon > 0$.

A compact manifold with a family of metrics as in the Definition 2 with $\mathrm {sec}$ changed to ${\mathrm{Ric}}$ is a manifold with *almost non-negative Ricci curvature*. As a last result in this section we prove:

#### Theorem 4.5

Let $F\hookrightarrow M \rightarrow B$ be a fiber bundle with compact structure group *G* and total space *M*. Also assume that *F* carries a metric $\textit{g}_F$ of non-negative Ricci curvature and *B* carries a metric $\textit{g}_{\epsilon }$ with ${\mathrm{Ric}}(\textit{g}_{\epsilon }) \ge -\epsilon ^2$. Then *M* carries a metric $\textit{h}_{\epsilon }$ with ${\mathrm{Ric}}(\textit{h}_{\epsilon }) \ge -\epsilon ^2$.

#### Proof

Let $\textit{h}_{\epsilon }$ be the connection Riemannian metric on *M*. That is, it has totally geodesic fibers and make $(M,\textit{h}) \rightarrow (B,\textit{g}_{\epsilon })$ to be a Riemannian submersion. Considering the $\textit{h}^{\epsilon }_t$ deformation given by Definition 1 we see that for large *t* it holds that, according to Lemma 7, the limit behavior of ${\mathrm{Ric}}(h^{\epsilon }_t)$ is

44 $$\begin{aligned} {\mathrm{Ric}}_{\bar{\textit{g}}}(d\pi X) + {\mathrm{Ric}}^{\mathbf {h}}(X_F) + 3\sum _{j=1}|A^{\pi _F}_{X_F}e_j^F|_{g_F}^2 + \sum _k\frac{1}{4}\Vert [v_k(0),U]\Vert _Q^2 \ge -\epsilon ^2. \end{aligned}$$

$$\square $$

## Some comments on the Petersen–Wilhelm fiber dimension conjecture

Related to the Petersen–Wilhelm conjecture, assume that $S^3, SO(3) \hookrightarrow \mathcal P \rightarrow B$ is a principal bundle with positive sectional curvature. It is then conjectured that $\dim B > 3$. We conjecture further:

### Conjecture 5.1

(Principal bundle Strong Petersen–Wilhelm conjecture) Any $S^3, SO(3)$ principal bundle over a positively curved manifold admits a metric with positive sectional curvature if, and only if, such a submersion is fat.

Assume for instance the validity of Conjecture 5.1 and take a fat principal bundle $S^3, SO(3) \hookrightarrow \mathcal P \rightarrow B$. Regard it with a metric of positive sectional curvature. Now let $(F,\textit{g}_F)$ be a Riemannian manifold with a $S^3, SO(3)$ isometric action. Assume that $\textit{g}_F$ has positive sectional curvature. Then Theorem 3.1 implies that the $\textit{h}_1$ metric deformation (Definition 1) has non-negative sectional curvature. More importantly, Remark 1 implies that the existence of flat planes is *intrinsic* in the sense it only depends on the $G = S^3, SO(3)$ actions on both $\mathcal P$ and *F*.

Now recall that if $F = S^2$ and $\mathcal P \rightarrow B$ is the *SO*(3) principal bundle associated to it, then $\overline{\pi }: S^2 \hookrightarrow M \rightarrow B$ is fat if, and only if, $\pi : SO(3)\hookrightarrow \mathcal P \rightarrow B$ is fat ([26, Proposition 2.22, p.16]). This implies that $\dim B \ge 4$ and hence, Petersen–Wilhelm conjecture is verified in this case. More drastically, the existence of a metric of non-negative sectional curvature on *M* already verifies the conjecture.

That all said and also taking in account the results in [17], we are tempted to conjecture the following:

### Conjecture 5.2

Let $\overline{\pi }: F\hookrightarrow M \rightarrow B$ a fiber bundle with structure group $S^3$ or *SO*(3) over a positively curved manifold *B*. If the principal bundle $\pi : S^3, SO(3) \hookrightarrow \mathcal P \rightarrow B$ associated to it is fat and $\overline{\pi }$ has a metric of positive vertizontal curvature then $\overline{\pi }$ is fat.

Let us verify that this is precisely the case to $S^2$-fat bundles, therefore agreeing with our conjecture. More precisely, let us show that $\textit{h}_1$ has positive vertizontal curvature.

Observe that the vertical space associated to $\overline{\pi }$ consists of vectors tangent to $F = S^2$. Since the *SO*(3) action on $S^2$ is transitive and $S^2$ can be identified with the homogeneous space *SO*(3)/*SO*(2) then for the fixed origin $o\in F$ we have $T_0F \cong \mathfrak {s}o(3)\ominus \mathfrak {s}o(2)$, meaning that $T_oF$ is isomorphic to the complement of $\mathfrak {s}o(2)$ in $\mathfrak {s}o(3)$. Fixing an Ad-invariant inner product in $\mathfrak {s}o(3)$ such a complement can be chosen to be orthogonal.

Finally, the horizontal space on $\mathcal P$ at any $p\in \mathcal P$ is isomorphic to the tangent space $T_{\pi (p)}B$ and it is also isomorphic to the horizontal space orthogonal to $T_oF$ with respect to $\textit{h}_1$. That is,

$$\begin{aligned} T_{[(p,o)]}M \cong T_oF\oplus \mathcal H^{\pi } \cong (\mathfrak {s}o(3)\ominus \mathfrak {s}o(2))\oplus (\mathfrak {s}o(3))^{\perp _{\textit{g}}}. \end{aligned}$$

Therefore, any vertizontal plane tangent to *M* is of the form $U^*\wedge X$ for $U\in \mathfrak {s}o(3)\ominus \mathfrak {s}o(2)$ and $X\in (\mathfrak {s}o(3))^{\perp _{\textit{g}}}$. Therefore Theorem 3.1 implies that

$$\begin{aligned} \widetilde{K}_1(X,U^*) \ge K_{\textit{g}_1}\left(X,U^{\vee }\right) > 0 \Leftrightarrow U^{\vee } \ne 0. \end{aligned}$$

Since the *SO*(3) action on $\mathcal P$ is free we have concluded the result. $\square$
